# Methodology of the development of the EULAR Antiphospholipid Syndrome Disease Activity Score (EAPSDAS)

**DOI:** 10.1016/j.ero.2025.07.009

**Published:** 2025-11-19

**Authors:** Maria G. Tektonidou, Ricard Cervera, Angela Tincani, Guillermo J. Pons-Estel, Savino Sciascia, Tanja Stamm, Robert B.M. Landewé, Michael M. Ward

**Affiliations:** 1 Rheumatology Unit, First Department of Propaedeutic Internal Medicine, Joint Academic Rheumatology Program, Medical School, National and Kapodistrian University of Athens, Athens, Greece; 2 Department of Autoimmune Diseases, Hospital Clínic, IDIBAPS, University of Barcelona, Barcelona, Catalonia, Spain; 3 Rheumatology and Clinical Immunology Unit-ERN ReCONNET, ASST-Spedali Civili and University of Brescia, Brescia, Italy; 4 Department of Rheumatology, Grupo Oroño-Centro Regional de Enfermedades Autoinmunes y Reumáticas (GO-CREAR), Rosario, Argentina; 5 University Center of Excellence on Nephrologic, Rheumatologic and Rare Diseases (ERK-Net, ERN-Reconnet and RITA-ERN Member), University of Turin, Turin, Italy; 6 Institute for Outcomes Research, Center for Medical Data Science, Medical University of Vienna, and Ludwig Boltzmann Institute for Arthritis and Rehabilitation, Vienna, Austria; 7 Department of Rheumatology and Clinical Immunology, Amsterdam University Medical Center, Amsterdam Rheumatology Center (ARC), Amsterdam, Netherlands, and Zuyderland Medical Center, Heerlen, Netherlands; 8 Verier Outcomes Research, Rockville, Maryland, USA

## Abstract

**Objectives:**

To describe the methodology of the development of a EULAR disease activity score in antiphospholipid syndrome (EAPSDAS).

**Methods:**

In phase I/item generation, we used a systematic literature review (SLR) and 2 open-ended surveys among Task Force members, experts in antiphospholipid syndrome (APS)/collaborators, patients with primary APS, and health professionals in rheumatology (HPRs). In phase II/item reduction, similar items were eliminated, participants rated items on their importance to be included in EAPSDAS, and the Delphi methodology/voting was used. In phase III/Item scoring, we used real-world clinical vignettes to develop scores for included items. We applied linear regression analysis with physician global assessment (PhysGA) as the standard.

**Results:**

SLR retrieved 3834 articles; 47 were analyzed. Of 170 items generated from the SLR and initial surveys, 140 deduplicated candidate items were rated by 74 physicians, 65 patients, and 21 HPRs and voted on by Task Force members. Twenty-four items were included in the EAPSDAS thrombotic/microvascular/nonthrombotic (TMN) scale, and 6 in the obstetric scale. Three versions of 60 real-world clinical vignettes were randomly assigned to participating physicians, split into 3 groups. The items’ score was calculated as the mean PhysGA adjusted for physician characteristics. Pairwise ranking comparisons analysis supported the mean vignette rankings. Analysis of ratings of the combinations of single manifestations showed no significant enhancement in scores except for 6 of 26 combinations of manifestations, supporting a ‘bump-up’ in these scores. TMN manifestations that should be scored if they remained stable or improved were determined by a survey.

**Conclusions:**

EAPSDAS was developed using data-driven and consensus methodology.

## INTRODUCTION

Antiphospholipid syndrome (APS) is a devastating autoimmune disorder with a complex pathogenesis and clinical presentation, as well as heterogeneous management [[1], [2], [3], [4], [5], [6]]. APS was first described 4 decades ago as a syndrome characterized by venous and/or arterial thrombosis and recurrent abortions in association with the presence of antiphospholipid antibodies (aPLs) [7]. Since then, advances in our understanding of its clinical phenotypes and disease pathophysiology [[8], [9], [10], [11], [12], [13], [14], [15], [16]] have helped identify potential new treatment targets. The development of well-designed clinical studies in APS would be facilitated by specific validated outcome measures [17].

Our goal was to develop a EULAR APS Disease Activity Score (EAPSDAS), a numerical tool for quantifying and assessing disease activity in APS in clinical practice, observational studies, and clinical trials. The current manuscript accompanies the main manuscript [18] and describes in detail the methodology used to develop the EAPSDAS and key results.

## METHODS

### Task Force

The project procedure was based on the EULAR standardised operating procedure. The Task Force included participants from 13 countries: 18 physicians of diverse clinical specialties (including 2 EMEUNET members), 3 methodologists, 1 nonphysician health professional in rheumatology (HPR), and 2 patient research partners.

### Methodology

The convenor (MGT), methodologists (RBML, TS, and MMW), and steering committee members (RC, AT, GJP-E, and SS) developed an outline of the project methodology. As in previous disease activity scores in rheumatic diseases, the methodology of developing the EAPSDAS included the item generation (phase I), item reduction (phase II), and item scoring using clinical vignettes (phase III).

Surveys were developed in all phases of the project using the SurveyMonkey platform, which is general data protection regulation compliant. Participation for patients and HPRs was anonymous, and survey access based on IP address was restricted for all. When responding to surveys, participants were asked to consider a patient with primary APS to avoid confounding related to manifestations shared between APS and other autoimmune disorders, eg, systemic lupus erythematosus (SLE). It was also indicated that disease activity should be rated based on the development of a new manifestation or worsening of a previous manifestation while also considering its specificity for the syndrome.

We began with several general principles:

1. To create a score for thrombotic, microvascular, and non-thrombotic (TMN) APS manifestations and a separate score for obstetric manifestations. Although the former may be applied to pregnant patients with primary APS, the derivation of each score will be separate. This separation was necessary because pregnancy occurs in only some patients and for a limited time. Integrating scores for pregnancy-related APS events and non–pregnancy-related events would result in a score potentially dominated by pregnancy-related events.
2. Ratings for individual manifestations would be based primarily on the regression analysis results of their relationship with the physician’s global activity assessment. Ratings will form the basis of the overall scores but would not be determinative, and final ratings may be adjusted by the steering committee/methodologists based on clinical rationale and other data inputs.
3. Responses from physicians who have >10% missing responses will be omitted.
4. The format of the scores may use the sum of ratings of individual manifestations, the highest rating overall, or a combination of these approaches. This decision may also be affected by the consideration that most new manifestations of APS occur singly, and it is rare to observe the simultaneous presentation of 2 new manifestations, or to have different organ systems active simultaneously, as in SLE.
5. Additional rounds of physician surveys may be used to resolve issues regarding rankings (eg, resolve ties or confirm similarity).
6. The preliminary score developed using the current data will be tested for initial reliability and validity in a manner determined by the steering committee/methodologists.

### Phase I. Item generation

Our goal was to develop a list of all potential candidate items associated with APS disease activity using 2 surveys among APS experts and patients and using a systematic literature review (SLR).

#### First survey

The first survey included an open-ended question to characterize and define the concept of disease activity in primary APS. Separate surveys were sent to physicians and patients. The question to the Task Force members and collaborators was as follows: ‘What’s the concept that in your experience indicates “disease activity” in primary antiphospholipid syndrome?’ Using patient-friendly language, the corresponding question asked to patients was: ‘If you wanted to tell your doctor that you believe your disease is in an active state or if you wanted to describe “disease activity” in primary APS to a caregiver or a friend, how would you explain this concept?’ Patients could respond to the 2 open-ended questions in English or other languages.

#### SLR

The outline of the proposed methodology and research questions for the SLR was defined in a meeting between the steering committee members and the methodologists (MMW and TS). The protocol for the literature search was prepared by MMW and the convenor (MGT) based on the Preferred Reporting Items for Systematic Reviews and Meta-Analyses statement [19]. Inclusion criteria included studies reporting on APS manifestations related to disease activity, restricting the search to primary APS and adults. The exclusion criteria were case reports/series, protocols, letters, editorials, commentaries, conference abstracts/proceedings, reviews and meta-analyses, animal studies, laboratory/*in vitro* studies, pediatric studies, studies with <20 participants, studies not reporting whether patients fulfilled the classification criteria for APS, studies on aPL carriers not fulfilling the classification criteria for APS, studies not reporting on primary APS separately, selected populations, and unrelated topics. Search terms were developed with the aid of an experienced librarian at the National Institutes of Health, USA, who performed the searches in collaboration with MMW. The search terms are presented in Supplemental Methods (‘Search terms’).

The SLR was conducted by 2 fellows, 2 EMEUNET members, and 1 Task Force member with experience in SLRs and under the guidance of a methodologist (MMW) and convenor (MGT). PubMed and Embase databases were searched for full-length English-language published articles from their inception through November 1, 2021. All titles and abstracts of retrieved articles were reviewed independently by 2 groups, each consisting of 2 or 3 literature reviewers. The full-text articles were independently reviewed by both literature reviewers in each group. Data abstraction was performed according to a written protocol by the 2 groups of reviewers. One of 2 literature reviewers and the convenor independently double-checked data abstraction in each group. The main data of interest were the type and frequency of clinical manifestations that were present in patients with active APS.

#### Second survey

This survey consisted of an open-ended question: ‘Please describe all features - clinical, laboratory, radiological, histological - that in your experience indicate disease activity in antiphospholipid syndrome (please think about a patient with primary antiphospholipid syndrome)’.

### Phase II. Item reduction

We compared the lists of candidate items from physician and patient responses in phase I surveys and those retrieved by the SLR, and we eliminated similar or highly related items. Following this consolidation of items, a third survey was prepared asking participants (APS experts, patients, and HPRs) to ‘rate the items for how important they are for inclusion in a disease activity score for APS’, using an 11-point rating scale where 0 = not at all important and 10 = extremely important. Items were presented in random order in the survey. The following explanatory information was provided in the survey’s introduction: ‘Please consider that your answers for each item should be based on its specificity, clinical importance, and potential for improvement or reversibility’. We also included the following statement for the patients: ‘We understand that there are some questions, including medical terms you are not familiar with. You can leave these questions unrated’.

The ratings of deduplicated items from the 3 groups of participants (APS experts, patients, HPRs) were sent to all Task Force members 1 week before the Task Force meeting for their consideration and comments. A 2-day virtual Task Force meeting took place on December 6 and December 9, 2022. An introduction to the methodology of the project and the results of the SLR and the phase I surveys, focusing on item generation, was presented by the convenor. The methodologists (MMW and TS) further explained the process and the main objective of the meeting, which was the item reduction (phase II). This was followed by discussions among all the members on the concept of disease activity in APS and the list of items. Each item was then digitally voted upon for inclusion or exclusion. Task Force members were asked to vote (raise hand) on whether they agree to keep an item in the list of candidate items for a disease activity score in APS, based on its specificity to identify disease activity, importance, potential response to treatment, frequency, and feasibility.

### Phase III. Item scoring

#### A Clinical vignettes

Following consensus, the methodology of phase III included the use of the clinical vignette exercise.

Real-world clinical vignettes were abstracted from the medical files of the steering committee member centers after approval of the project by their institutional review board. Each vignette was based on a patient who had newly developed or had worsening of 1 of the target manifestations over the past month. Three different patients were abstracted for each new/worsening APS manifestation, with different background information (eg, demographics, past history, or comorbidities). This triplicate approach was used so that patients with different background characteristics would be rated, reducing the chance that any specific background characteristic could influence the ratings.

Each clinical vignette contained the following sections: ‘APS history’ (demographic data and medical history), ‘Current presentation’ (clinical symptoms and results of imaging or histologic examination), and ‘Labs’ (laboratory examinations) (Supplemental Methods, ‘Clinical vignette examples’). Participants were asked to rate the level of current disease activity for each of the clinical vignettes using the rating scale (0 = none; 10 = extremely active). To assess the representativeness of the vignettes, participants were also asked the following question: ‘In your experience, how likely would you see a patient with a similar presentation in your practice? (1) typical, (2) somewhat typical, and (3) not typical (check one)’. A list of potential combinations of 2 items (the so-called ‘double manifestations’) among the TMN features was developed. The committee thought that it would be very rare for a patient to develop 3 new APS manifestations concurrently, so vignettes for such cases were not developed. No double manifestations were thought likely among the Obstetric items.

Three survey versions (A, B, and C) were created in the SurveyMonkey, each including single-item cases with 1 new/worsening manifestation, 1 case with thrombotic APS history at a regular visit without any new/worsened features, 1 case with uncomplicated pregnancy, and double-manifestation cases. Each survey also included 2 practice vignettes (testing cases) to orient the respondents to the rating task. Physicians from the Task Force group and collaborators were randomly divided into 3 groups, each of which received 1 vignette version.

Within each survey, testing vignettes for practice were presented first, but the remaining vignettes were presented in random order. Therefore, each rater received the clinical vignettes in a different order. An example of 3 vignette versions of a single manifestation (venous thromboembolism [VTE] in 1 territory) is presented in Supplemental Methods (‘Clinical vignette examples’).

#### B Statistical analysis

Analysis proceeded in several steps. As an initial check on the vignette responses, we assessed representativeness by computing the frequency of vignettes that were rated as very typical, typical, or atypical by the physician raters. We next tested if ratings of the divided items (moderate vs severe thrombocytopenia; nonnephrotic vs nephrotic-range proteinuria; unprovoked vs provoked venous thromboembolism) were significantly different using paired *t* tests and rank-sum tests. These results indicated whether these distinctions should be retained or if these items should be collapsed into single items.

The main analysis calculated a score for each item based on its average rating in the physician global assessment (PhysGA; 0-10) of the vignettes describing new/worsening manifestations. Items in the TMN scale were analyzed separately from those in the Obstetric scale. Linear regression models were used to derive adjusted mean scores, with adjustment for physician age (35-44/45-54/55-64/65 years or older), physician gender, geographic region (Europe/North America/Rest of world), rheumatologist vs other specialty, years in practice (0-9/10-19/20 years or more), average number of patients seen monthly (0-4/5-9/10-19/20 patients or more), and vignette version (A, B, or C).

Because mean scores may be skewed by a small proportion of extreme values, we checked if the adjusted mean scores were consistent with the rank order of the majority of physician raters. We first defined all pairs of single items in the TMN and Obstetric scales. For each pair of items, we identified the proportion of physicians who rated item A higher than item B or vice versa. We then compared these proportions with the ranking provided by the adjusted mean scores to determine if these rankings were consistent. For example, for the pair ‘one arterial event/skin ulcer’, we counted the proportion of physicians who rated the patients with ‘one arterial event’ as more active than the patient with ‘skin ulcer’, the proportion who rated the patient with ‘skin ulcer’ higher, and the proportion who rated these the same. We then compared whether these proportions were aligned with the adjusted mean scores of ‘one arterial event’ and ‘skin ulcers’. This paired comparison procedure was repeated for 276 pairs of TMN items and 15 pairs of Obstetric items. Next, we examined if modifications to the average scores were indicated when 2 manifestations newly occurred or worsened together, using data provided from the vignettes describing such patients. We first computed the difference in PhysGA ratings between the vignettes with 2 new/worsening manifestations (eg, new stroke and new livedo reticularis) and the PhyGA of vignettes where each component manifestation occurred singly (eg, new stroke; new livedo reticularis). We then tested these double-manifestation ratings in linear regression models to estimate the additional activity score that reflected the co-occurrence of the second manifestation. For those double-manifestation vignettes that had significantly higher PhysGAs than the higher-rated single-item vignette of the pair, we used the adjusted mean difference to estimate a ‘bump-up’ in score when these manifestations co-occurred.

#### C Longitudinal scoring considerations

A second Task Force meeting took place on the February 20, 2024. The following 3 main issues were discussed: (1) the time frame for TMN and Obstetric scale manifestations, (2) how much time should have elapsed before a second venous or arterial event would be counted as ‘new’ APS activity, rather than ‘continuation’ of the previous activity, and (3) whether and how ‘abnormal but stable’ (eg, persistent moderate thrombocytopenia or nonnephrotic proteinuria) or ‘improved but still present’ manifestations should be scored. No consensus was reached. After further discussions with the steering committee and the methodologists, and after receiving the agreement from all Task Force members (by email), a new survey was developed and sent to physicians/experts from the Task Force group and the collaborators.

For the time interval of venous or arterial thrombotic events, the question was ‘How long should an arterial (or venous) thrombosis be considered active after its first presentation?’ with the following answer choices: up to 1 month, 2 months, 3 months, and other (please specify). For the scoring of stable or improved manifestations, some example questions are the following: ‘Should severe thrombocytopenia be scored if it is new or worsened?’, ‘Should severe thrombocytopenia be scored if it is abnormal but stable?’, and ‘Should severe thrombocytopenia be scored if it is improved but still present?’. ‘If you voted YES that “improved but still present” severe thrombocytopenia should be scored, should the original score or a modified one be used?’.

## RESULTS

### Participants

Fifty-six experts in APS were invited by the convenor (MGT) to participate in the project exercises as collaborators. Fifty-three (94.6%) individuals from Europe, North America, South America, Asia, and Australia/Oceania accepted the invitation. Among 74 participants in the surveys (Task Force members excluding the 3 methodologists, and collaborators), the majority were rheumatologists (57%), internists (11%), and hematologists and vascular medicine doctors (9%), followed by obstetricians, immunologists, cardiologists, and pathologists.

An international group of 90 patients with primary APS was accepted to participate anonymously in the phase I and II surveys. Patients were recruited via EULAR PARE, APS UK, APS Spain, and the European Reference Network on Rare and Complex Connective Tissue and Musculoskeletal Diseases (ERN ReCONNET) APS group. In addition, 25 HPRs (nurses and physical and occupational therapists) with experience in APS were contacted via the Task Force members, and 21 (84%) agreed to participate in phase II: 76% were from Europe, and the rest from North America, South America, and Asia.

### Phase I. Item generation

#### First survey: concept of activity in APS

In total, 21 Task Force members, 53 international APS experts, and 90 patients agreed to participate. Seventy-one of 74 Task Force members and collaborators defined the concept of disease activity in APS mainly by clinical manifestations: both ‘criteria’ (thrombotic and obstetric manifestations) and the previously called noncriteria APS manifestations (microvascular/cardiac/hematological/nonthrombotic manifestations) (45/74), criteria APS manifestations only (21/74), and noncriteria APS manifestations only (5/74). There was a variable emphasis on laboratory tests of different types: inflammatory markers (20/74), aPL titers or aPL profile (15/74), and coagulation markers (eg, D-dimers) (8/74). Regarding disease activity qualifiers, 30 physicians reported new or acute events, 22 recurrent or worsened events, 7 modifiable events after treatment, and 4 relapses.

Among the 95 patients who participated in the survey, 65 had complete responses: 87% were women, their mean age was 44.8 years (20-76 years), 81.5% were from Europe, and the rest were from North America, South America, and Australia/Oceania. Among 65 patients with complete data, 8 reported on worsening events, 7 on modifiable features, 8 on 0 manifestations, 5 on new or acute, and 1 on recurrent events.

#### SLR results

A total of 3834 articles were identified in the SLR, and 47 articles were ultimately included in the full-text review (Fig). After excluding similar manifestations, 77 candidate items that reflected APS activity were identified and classified into clinical, laboratory, radiologic, and histologic domains (Supplementary Table S1). The clinical domain included the classic thrombotic and obstetric manifestations of APS and the noncriteria manifestations, as defined by the previous APS criteria, since the SLR was conducted before the publication of the new 2023 ACR/EULAR APS classification criteria [3].

**Figure 1:**
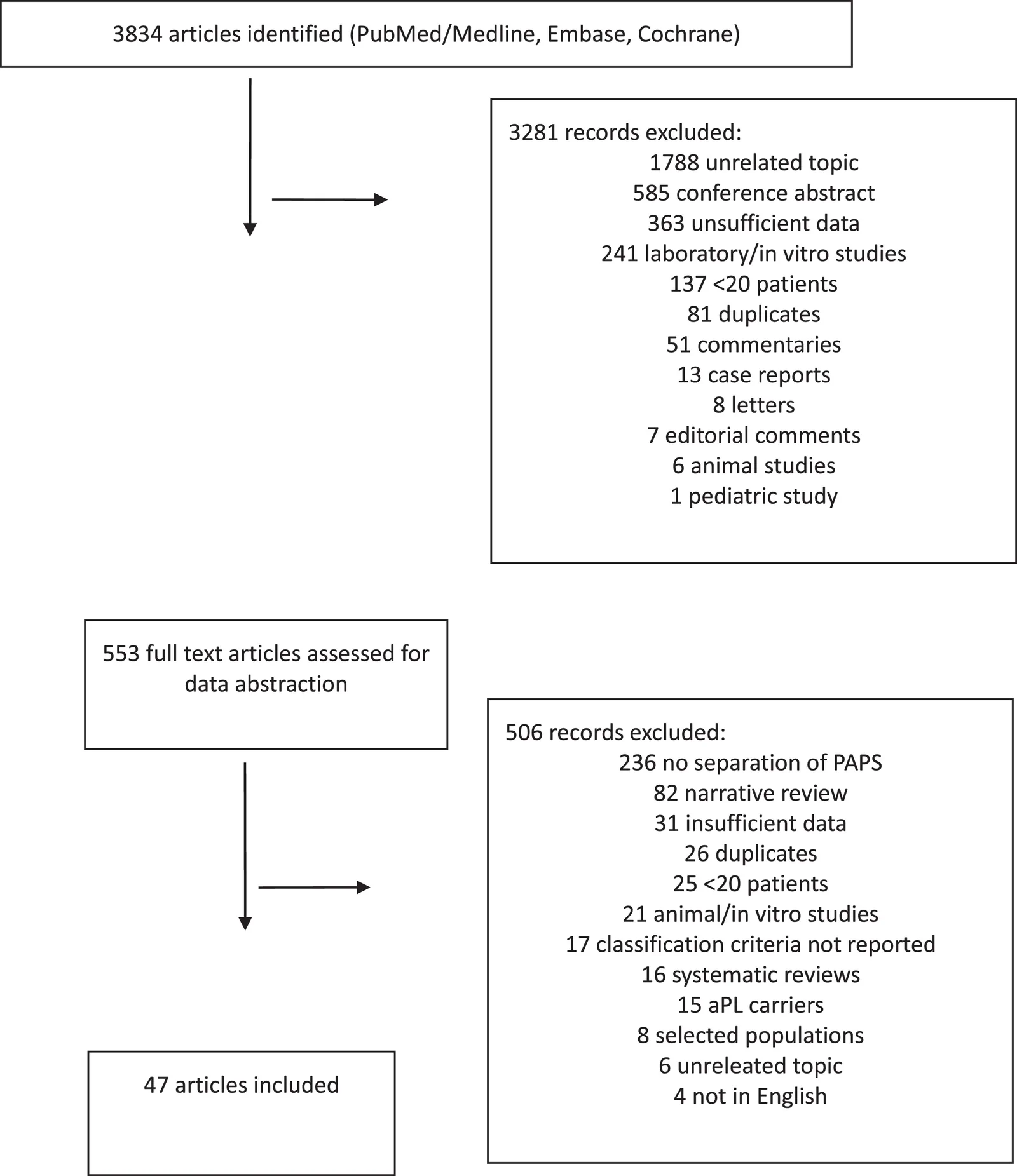
Flow chart for the systematic literature review.

#### Second survey: items representing activity

In the second survey, 21 Task Force members, 52 international experts, and 59 patients participated. Regarding the second survey results, 132 items were identified from the physicians’ responses and 59 from patients’ responses. In total, 170 unique items were generated by the SLR and the surveys. The items retrieved by the SLR (*n* = 77), physicians’ responses (*n* = 132), and patients’ responses (*n* = 59) are presented in Table 1, ordered by the physician’s mean importance in the third survey.

**Table 1:** Items retrieved from the systematic literature review and the second survey results. Items are ordered by physicians’ mean importance in the third survey (shown in Table 2)

Items	Found in the literature review (yes:1, 0: no, NA: not applicable)	Mentioned by physicians in the second survey (yes: 1, 0: no)	Mentioned by patients in the second survey (yes: 1, 0: no)	Retained for voting, based on the physician’s mean importance (yes: 1, 0: no)	Endorsed by >50% of the Task Force members (yes: 1, 0: no)	Endorsed by >75% of the Task Force members (yes: 1, 0: no)
1. Catastrophic APS	1	1	1	1	1	1
2. Pulmonary embolism	1	1	1	1	1	0^a^
3. Arterial thrombosis	1	1	1	1	1	1
4. Deep vein thrombosis	1	1	1	1	1	0^a^
5. Ischemic stroke	1	1	1	1	1	0^a^
6. Splanchnic venous thrombosis	1	1	0	1	1	0^a^
7. Splanchnic artery thrombosis	1	1	0	1	1	0^a^
8. Microangiopathy, microvascular thrombosis	1	1	0	1	1	0^a^
9. Cerebral venous sinus thrombosis	1	1	0	1	1	0^a^
10. Radiologic signs of thrombosis peripheral artery or vein, organs (brain, heart, splanchnic)	1	1	0	1	1	0^a^
11. Severe pre-eclampsia/eclampsia	1	1	0	1	1	1
12. Histologic signs of microthrombi or thrombotic microangiopathy (eg, renal, skin)	1	1	0	1	1	0^a^
13. Venous thrombosis	1	1	1	1	1	1
14. Obstetric complications	1	1	1	1	0	0
15. APS nephropathy	1	1	0	1	1	1
16. Myocardial infarction	1	1	1	1	1	0^a^
17. Thrombocytopenia	1	1	0	1	1	1
18. Triple-positive antiphospholipid antibodies	1	1	0	1	1	0
19. Intracardiac thrombi on imaging	1	1	0	1	1	0^a^
20. Ophthalmic arterial or venous thrombosis	1	1	0	1	1	0^a^
21. Fetal death unexplained	1	1	1	1	1	1
22. Extensive villous ischemia, villitis, and utero-placental vasculitis, multifocal utero-placental thromboses	1	1	0	1	1	0^a^
23. Lupus anticoagulant	1	1	0	1	0	0
24. Hemolytic anemia (autoimmune or microangiopathic)	1	1	0	1	1	1
25. Placental insufficiency	1	1	0	1	0	0
26. Persistently high/increase in anti-β2GPI antibody titers	1	1	0	1	1	0
27. Brain ischemic lesions on magnetic resonance imaging	1	1	0	1	1	0^a^
28. Cardiac valve vegetations	1	1	0	1	1	1
29. Digital ischemia/gangrene	1	1	0	1	1	1
30. Skin ulcers	1	1	0	1	1	1
31. Transient ischemic attack	1	1	0	1	1	0
32. HELLP syndrome	1	1	0	1	1	1
33. Persistently high/increase in titers of anticardiolipin antibodies	1	1	0	0	1	0
34. Cardiac microvascular disease on magnetic resonance imaging	1	1	0	1	1	0
35. Intrauterine growth restriction (IUGR)	1	1	0	1	1	1
36. New recurrent first-trimester miscarriage	1	1	1	1	1	1
37. Diffuse alveolar hemorrhage	1	1	0	1	1	1
38. Newly positive antiphospholipid antibodies	1	1	0	1	0	0
39. Adrenal hemorrhage	1	1	0	1	1	1
40. Transverse myelitis	1	1	0	1	1	1
41. Livedoid vasculopathy	1	1	0	1	1	1
42. Livedo reticularis or livedo reticularis	1	1	1	1	1	1
43. Acute organ dysfunction (kidney, heart, lung)	1	1	0	1	0	0
44. Low complement levels	1	1	0	1	0	0
45. Chorea	1	1	0	1	0	0
46. Amaurosis fugax	1	1	0	1	1	0
47. Noninfectious endocarditis (Libman-Sacks)	1	1	0	1	1	0^a^
48. Elevation in liver enzymes due to hepatic thrombosis	1	1	0	1	0^a^	0^a^
49. Antiprothrombin antibodies	NA	1	0	1	0	0
50. Angina	1	1	0	1	1	0^a^
51. Pulmonary hypertension	1	1	0	1	1	1
52. Multiple sclerosis–like symptoms	1	1	0	1	0	0
53. Epilepsy, new seizures	1	1	0	1	1	1
54. Proteinuria	1	1	0	1	1	0^a^
55. Cognitive/memory impairment	1	1	1	1	1	0
56. Renal insufficiency	1	1	0	1	1	0^a^
57. Premature birth due to placental insufficiency	1	1	0	1	1	1
58. Elevated D-dimers	1	1	0	1	0	0
59. High global antiphospholipid syndrome score (aGAPSS)	NA	1	0	0	0	0
60. Superficial venous thrombosis	0	1	0	1	1	0
61. Avascular necrosis/osteonecrosis	1	1	0	1	1	1
62. Uterine doppler abnormality	NA	1	0	1	0	0
63. Cardiac valve thickening	1	1	0	1	1	1
64. Heart strain on imaging	0	1	0	1	0	0
65. Platelet activation markers	NA	1	0	1	0	0
66. Adult respiratory distress syndrome	NA	1	0	1	0	0
67. Cytopenia	NA	1	0	1	0	0
68. Elevated complement split products	NA	1	0	1	0	0
69. Hematuria	1	1	0	1	0	0
70. Endothelial activation	NA	1	0	1	0	0
71. Heart failure due to cardiomyopathy	1	1	0	0	0	0
72. Sensorineural hearing loss	1	1	0	1	0	0
73. Splinter hemorrhages	1	1	0	1	0	0
74. Histologic signs of endothelial proliferation	NA	1	0	1	0	0
75. Prior poor obstetric outcomes	NA	1	0	1	0	0
76. sFLT1/PIGF ratio	NA	1	0	1	0	0
77. Longitudinal myelitis	1	1	0	1	1	0
78. Systemic inflammatory reaction	NA	1	0	1	0	0
79. Oligohydramnios	1	1	0	1	0	0
80. Portal hypertension	1	1	0	1	0	0
81. Multiple sclerosis–like lesions on magnetic resonance imaging	1	1	0	1	0	0
82. Activated protein C resistance	NA	1	0	1	0	0
83. Raynaud's phenomenon	NA	1	0	1	0	0
84. Migraine	1	1	1	1	1	
85. Low INR	NA	1	1	1	0	0
86. Brain fog	NA	0	1	1	0	0
87. Enhanced netosis	NA	1	0	1	0	0
88. Cardiovascular risk factors	NA	1	1	1	0	0
89. Shortness of breath	NA	0	1	1	0	0
90. Encephalopathy	NA	1	0	1	0	0
91. Hypertension	NA	1	0	1	0	0
92. New immunological disease	NA	0	1	1	0	0
93. Transient global amnesia	1	1	0	1	0	0
94. Elevated ferritin levels	NA	1	0	1	0	0
95. Elevated prothrombin fragments	NA	1	0	1	0	0
96. Acute anemia	NA	1	0	1	0	0
97. Pulmonary edema	NA	1	0	1	0	0
98. Dementia	1	1	0	1	0	0
99. Intracranial hypertension	NA	1	0	1	0	0
100. Elevated levels of C-reactive protein	NA	1	0	1	0	0
101. Guillain-Barre syndrome	1	1	0	1	0	0
102. Imbalance/dizziness	NA	0	1	1	0	0
103. Coexisting inherited thrombophilia	NA	1	0	1	0	0
104. Atheromatous plaques	NA	1	0	1	0	0
105. Family history of autoimmune disease	NA	0	1	1	0	0
106. Headache	NA	1	1	1	0	0
107. Psychosis	NA	1	0	1	0	0
108. Proinflammatory cytokines	NA	1	0	1	0	0
109. Leukopenia	NA	1	0	1	0	0
110. Fatigue	NA	1	1	1	0	0
111. Bruising	NA	0	1	1	0	0
112. Change in mood	NA	0	1	1	0	0
113. Ascites	NA	1	0	1	0	0
114. Bleeding	NA	0	1	1	0	0
115. Elevated erythrocyte sedimentation rate	NA	1	0	1	0	0
116. Recurrent acute pancreatitis	NA	1	0	1	0	0
117. Pain	NA	1	1	1	0	0
118. Arthralgias	NA	1	1	1	0	0
119. Genetic markers	NA	1	0	1	0	0
120. Depression	NA	1	1	1	0	0
121. Cardiovascular syndrome X	NA	1	0	1	0	0
122. Myalgias	NA	0	1	1	0	0
123. Elevated amylase, lipase	NA	1	0	1	0	0
124. Inflammatory arthritis	NA	1	0	1	0	0
125. Fever	NA	1	1	1	0	0
126. Paresthesia	NA	0	1	1	0	0
127. Skin nodules	NA	1	0	1	0	0
128. Swollen eyes	NA	0	1	1	0	0
129. Mobility issues	NA	1	0	1	0	0
130. Nodular regenerative hyperplasia	NA	1	0	1	0	0
131. Anti-Ro, anti-La antibodies	NA	1	0	1	0	0
132. Hyperactivity and behavioral abnormalities	NA	1	0	1	0	0
133. Elevated plasma lipoperoxides	NA	1	0	1	0	0
134. Swelling	NA	0	1	1	0	0
135. Dry eyes	NA	0	1	1	0	0
136. Menstrual changes	NA	0	1	1	0	0
137. Nausea	NA	0	1	1	0	0
138. Poor immune system	NA	0	1	1	0	
139. Heat flashes	NA	0	1	1	0	0
140. Skin allergy	NA	0	1	1	0	0
141. Limb ischemia, peripheral artery	1	1	0	1	0	0
142. Splenic infarct	1	1	0	0	0	0
143. Mesenteric ischemia or infarction	1	1	0	0	0	0
144. Budd-Chiari syndrome	1	1	0	0	0	0
145. Mitral or aortic regurgitation	1	1	0	0	0	0
146. Neurological involvement	NA	1	0	0	0	0
147. Elevated plasma lipoperoxides	NA	1	0	0	0	0
148. Dyslipidemia	NA	1	0	0	0	0
149. Loss of sight due to occlusion	NA	1	0	0	0	0
150. Kidney failure	NA	1	0	0	0	0
151. Clots on imaging	NA	0	1	0	0	0
152. Coagulation issues	NA	0	1	0	0	0
153. Brain problems	NA	0	1	0	0	0
154. Brain imaging abnormalities	NA	0	1	0	0	0
155. Poor circulation	NA	0	1	0	0	0
156. Calf pain and swelling	NA	0	1	0	0	0
157. Inflammation	NA	0	1	0	0	0
158. Skin manifestations	NA	0	1	0	0	0
159. Memory issues	1	0	1	0	0	0
160. Obstetric complications	NA	0	1	0	0	0
161. Premature delivery	NA	0	1	0	0	0
162. Frequent blood tests	NA	0	1	0	0	0
163. Mottled skin	NA	0	1	0	0	0
164. Heart inflammation	NA	0	1	0	0	0
165. Bruised fingers	NA	0	1	0	0	0
166. Inflamed veins	NA	0	1	0	0	0
167. Eye problems	NA	0	1	0	0	0
168. Vision loss	NA	0	1	0	0	0
169. Visual disturbance	NA	0	1	0	0	0
170. Ear symptoms	NA	0	1	0	0	0

aPL, antiphospholipid antibodies; APS, antiphospholipid syndrome; HELLP, hemolysis, elevated liver enzyme levels, and low platelet levels; HPRs, nonphysician health professional in rheumatology; MRI, magnetic resonance imaging; sFlt-1/PlGF, soluble fms-like tyrosine kinase-1/placental growth factor.

Items 141-170 were considered by the steering committee as duplicate items and were not discussed during the Task Force meeting/Delphi procedure.

a Items grouped with other items in a more general term, eg, pulmonary embolism is part of venous thromboembolism.

### Phase II. Item reduction

After deduplication of 170 items, 140 items remained and are presented in Table 1. In the third survey, these items were rated by 74 APS experts, 53 patients, and 21 HPRs based on their importance (0-10, with 10 being extremely important) to be included in an APS disease activity score (Table 2). The mean ± SD for each of 140 items was calculated separately for each of the 3 groups (physicians, patients, and HPRs) and is presented in Table 2. A summary table with these ratings was sent to all Task Force members 1 week before the Task Force meeting for their consideration and comments.

**Table 2:** Physicians’, health professionals’, and patients’ mean rankings from the third survey (items are ordered by physicians’ mean importance)

Manifestations	Physicians		HPRs		Patients	
	Mean	SD	Mean	SD	Mean	SD
1. Catastrophic APS	9.7	0.87	8.9	1.9	9.2	2.19
2. Pulmonary embolism	9.2	1.43	8.3	1.89	9.2	1.67
3. Arterial thrombosis	9.2	1.28	8.05	2.23	8.4	2.55
4. Deep vein thrombosis	9.1	1.27	7.8	2.1	9.1	1.64
5. Ischemic stroke	9.1	1.67	8.2	2.44	8.9	2.37
6. Splanchnic venous thrombosis	9.01	1.68	8.2	2.12	8.1	2.67
7. Splanchnic arterial thrombosis	9	1.35	7.7	2.59	7.7	3.06
8. Microangiopathy, microvascular thrombosis	9	1.28	7.5	2	7.4	2.88
9. Cerebral venous sinus thrombosis	8.9	1.71	8.1	2.03	8.4	2.57
10. Radiologic signs of thrombosis	8.9	1.6	8.06	2.19	8.9	1.48
11. Severe pre-eclampsia	8.8	1.34	7.7	2.09	7.9	2.92
12. Histologic signs of microthrombi or thrombotic microangiopathy (eg, renal, skin)	8.8	1.6	8.3	1.61	7.4	2.44
13. Venous thrombosis	8.7	1.81	7.8	2.05	8.9	1.75
14. Obstetric complications	8.7	1.51	8.5	1.95	8.8	2.52
15. APS nephropathy	8.7	1.64	7.7	1.8	8.3	2.75
16. Myocardial infarction	8.6	1.79	7.7	2.58	8.4	2.66
17. Thrombocytopenia	8.4	1.36	7.5	1.65	7.7	2.49
18. Triple antiphospholipid antibody positivity	8.4	2.37	8.75	1.52	9.09	1.82
19. Intracardiac thrombi	8.4	1.55	7.8	1.7	8.3	2.43
20. Ophthalmic arterial or venous thrombosis	8.3	1.79	7.8	2.44	8.2	2.62
21. Fetal death unexplained	8.3	2.14	8.2	2.18	8.6	2.78
22. Extensive villous ischemia, villitis, and utero-placental vasculitis, multifocal utero-placental thromboses	8.22	1.9	7.73	1.28	7.59	2.99
23. Lupus anticoagulant	8.2	2.39	8.8	1.45	8.9	1.7
24. Hemolytic anemia	8.04	1.52	6.9	2.13	7.1	2.38
25. Placental insufficiency	7.9	1.57	6.12	2.39	7.75	2.95
26. Persistently high/increase in anti-β2GPI antibody titers	7.9	2.45	8.1	1.7	8.1	2.22
27. Brain ischemic lesions on magnetic resonance imaging	7.9	1.9	7.75	1.52	8.52	2.26
28. Cardiac valve vegetations	7.8	2.03	6.1	1.91	6.7	2.98
29. Digital ischemia/gangrene	7.8	2.03	6.6	2.32	7.1	2.81
30. Skin ulcers	7.7	1.87	6.05	1.9	6.8	2.33
31. Transient ischemic attack	7.7	1.93	7.6	1.93	8.9	2.02
32. HELLP syndrome	7.7	2.26	6.2	3.08	7.7	2.7
33. Persistently high/increase in anticardiolipin antibody titers	7.7	2.41	8.2	1.78	8.1	2.69
34. Cardiac microvascular disease on magnetic resonance imaging	7.66	1.95	7.6	1.4	8.04	2.4
35. Intrauterine growth restriction (IUGR)	7.6	1.99	5.8	2.4	7.5	3.1
36. New recurrent first-trimester miscarriage	7.5	2.78	8	2.16	8.8	2.47
37. Diffuse alveolar hemorrhage	7.5	2.58	6.5	3.27	7.8	2.88
38. Newly positive antiphospholipid antibodies	7.4	2.21	8	1.31	8.4	2.53
39. Adrenal hemorrhage	7.3	2.48	5.8	2.95	7.3	2.84
40. Transverse myelitis	7.2	2.54	5.4	3.11	6.7	2.9
41. Livedoid vasculopathy	7.04	2.32	5.2	2.75	6.7	2.08
42. Livedo reticularis or livedo racemosa	7	2.04	6	2.19	6.3	2.51
43. Acute organ dysfunction (kidney, heart, lung)	7	2.85	6.25	2.9	7.4	3.04
44. Low complement levels	6.91	2.41	5.2	2.17	7.45	2.85
45. Chorea	6.8	2.53	4.8	2.68	6.2	2.68
46. Amaurosis fugax	6.8	3.04	5.5	2.66	5.7	3.5
47. Noninfectious endocarditis	6.8	2.27	5.3	2.73	6.6	3.01
48. Elevation in liver enzymes due to hepatic thrombosis	6.7	2.64	6.5	1.93	6.8	2.73
49. Antiprothrombin antibodies	6.61	2.62	6.4	2.93	7.8	2.3
50. Angina	6.6	2.46	8.7	2.17	7.08	3.12
51. Pulmonary hypertension	6.6	1.78	5.6	2.54	7.4	2.76
52. Multiple sclerosis–like lesions	6.6	2.46	5.6	2.46	7.92	2.33
53. Epilepsy/new seizures	6.4	2.4	5.9	2.79	6.8	2.5
54. Proteinuria	6.4	2.51	6.05	2.48	7.09	2.66
55. Cognitive/memory impairment	6.3	2.38	5.3	2.69	8.3	2.25
56. Renal insufficiency	6.3	2.52	6.11	2.76	7.2	2.87
57. Premature birth due to placental insufficiency	6.2	2.47	5.8	2	7	3.22
58. Elevated D-Dimers	6.2	2.52	5.8	2.94	7.4	2.8
59. High global antiphospholipid syndrome score (aGAPSS)	6.14	3.02	5.61	2.81	6.5	2.78
60. Superficial venous thrombosis	6.05	2.61	5.3	2.54	7.13	3.11
61. Avascular necrosis (osteonecrosis)	6.02	2.62	5.11	2.54	7.6	2.59
62. Uterine doppler abnormality	6	3.01	5.4	2.06	6.5	3.12
63. Cardiac valve thickening	5.9	2.84	2.2	2.41	7.7	2.63
64. Heart strain on imaging	5.9	2.38	5.6	2.82	8.04	2.22
65. Platelet activation markers	5.76	2.86	5	2.51	7	2.7
66. Adult respiratory distress syndrome	5.7	3.31	5	3.35	6.19	3.12
67. Cytopenia	5.7	2.81	5.4	2.22	6.4	2.8
68. Elevated complement split products	5.7	2.67	4.69	3.14	5.6	3
69. Hematuria	5.6	2.73	5.5	2.89	6.5	2.64
70. Endothelial activation	5.54	2.65	5.7	2.33	6.5	2.89
71. Heart failure due to cardiomyopathy	5.5	2.62	5.2	3.42	8.4	2.52
72. Sensorineural hearing loss	5.5	2.53	4.4	2.11	6.3	2.34
73. Splinter hemorrhages	5.5	2.75	5.6	2.68	6	2.78
74. Histologic signs of endothelial proliferation	5.5	2.82	3.8	2.47	5.6	2.94
75. Prior poor obstetric outcomes	5.5	3.41	6.2	2.33	7.2	3.04
76. sFLT1/PIGF ratio	5.4	3.04	5.18	1.9	6.08	2.78
77. Longitudinal myelitis	5.3	3.08	5.15	2.87	6.15	2.85
78. Systemic inflammatory reaction	5.26	2.82	5.64	2.71	7.2	2.59
79. Oligoydramnios	5.22	2.66	5.15	2.41	5.7	3.37
80. Portal hypertension	5.22	2.91	5.5	3.31	6.9	2.63
81. Multiple sclerosis–like symptoms	5.2	2.5	5.06	2.75	7.8	2.74
82. Activated protein C resistance	5.2	2.86	4.6	2.4	5.5	2.92
83. Raynaud's phenomenon	5.19	2.5	5.2	2.54	6.88	2.79
84. Migraine	5.1	2.57	4.7	2.23	7.8	2.3
85. Low INR	5.1	3.18	5.5	2.89	8.14	2.74
86. Brain fog	5.1	2.92	6.1	2.57	8.28	1.98
87. Enhanced netosis	5.08	2.7	4	2.6	5.41	3.06
88. Cardiovascular risk factors	5.07	3.14	5.8	2.24	7.55	2.23
89. Shortness of breath	5.07	2.96	5.3	3.14	7.4	2.62
90. Encephalopathy	5.07	2.97	4.93	3.25	6.35	2.68
91. Hypertension	5.04	2.51	4.5	2.17	7.8	2.48
92. New immunological disease	4.97	3.01	5.8	2.72	8.1	1.59
93. Transient global amnesia	4.9	3.07	4.5	2.82	6.8	3.08
94. Elevated ferritin levels	4.88	2.61	4.2	2.93	6.04	2.72
95. Elevated prothrombin fragments	4.88	2.78	5.3	2.49	6.3	2.8
96. Acute anemia	4.79	2.78	4.4	2.66	6.9	2.41
97. Pulmonary edema	4.7	2.89	5.21	3.32	7.3	2.57
98. Dementia	4.7	2.75	4.28	2.84	7.04	2.96
99. Intracranial hypertension	4.7	3.14	6.07	2.89	7.4	2.62
100. Elevated levels of C-reactive protein	4.61	2.96	5.73	2.79	6.5	2.77
101. Guillain-Barre syndrome	4.5	2.77	4.2	2.58	6.9	3.22
102. Imbalance/dizziness	4.5	2.8	4.9	2.3	8.09	2.38
103. Coexisting inherited thrombophilia	4.5	3.09	5.3	3.31	6.08	2.81
104. Atheromatous plaques	4.5	3.09	4.6	2.55	5.8	2.74
105. Family history of autoimmune disease	4.5	3.07	5	2.66	7.7	2.77
106. Headache	4.47	2.75	4.7	1.96	7.18	2.28
107. Psychosis	4.45	2.99	5.5	3.2	5.6	3.29
108. Proinflammatory cytokines	4.3	2.65	5.15	3.15	6.3	2.58
109. Leucopenia	4.29	2.87	5.5	2.44	6.07	2.84
110. Fatigue	4.2	2.87	5.3	2.9	8.3	1.89
111. Bruising	4.19	2.76	5.17	3.01	5.5	3.08
112. Change in mood	4.14	2.88	5.6	2.52	6.03	3.56
113. Ascites	4.12	2.8	4.78	2.93	6	2.78
114. Bleeding	4.1	3.29	5.4	2.77	7.2	2.75
115. Elevated erythrocyte sedimentation rate	4.07	2.6	5.07	2.4	6.3	2.91
116. Recurrent acute pancreatitis	4.05	2.83	5.07	3.12	6.14	3.07
117. Pain	3.9	2.76	5.6	2.69	8	1.68
118. Arthralgias	3.9	2.52	4.8	2.53	6.4	2.73
119. Genetic markers	3.73	3.03	5.4	3.27	7.5	2.85
120. Depression	3.7	2.67	5.8	2.88	5.8	3.59
121. Cardiovascular syndrome X	3.67	3.08	3.3	2.3	6.2	2.69
122. Myalgias	3.6	2.59	5.7	2.8	7.07	2.53
123. Elevated amylase, lipase	3.6	2.78	4.2	2.7	5.4	3.04
124. Inflammatory arthritis	3.5	2.6	4.4	3.07	7.2	2.6
125. Fever	3.4	2.91	4.8	3.45	5.03	3.21
126. Paresthesia	3.4	2.78	4.4	2.87	6.5	2.67
127. Skin nodules	3.36	2.71	3.07	1.94	5.5	2.83
128. Swollen eyes	3.32	2.63	4.3	2.86	4.8	2.84
129. Mobility issues	3.3	2.81	4.4	2.62	6.5	3.09
130. Nodular regenerative hyperplasia	3.17	2.55	3.5	2.06	6	2.82
131. Anti-Ro, anti-La antibodies	3.1	2.86	5.1	3.34	6.3	2.72
132. Hyperactivity and behavioral abnormalities	3.05	2.97	3.7	2.21	4.7	3.13
133. Elevated plasma lipoperoxides	3.04	2.61	3.6	2.05	5.6	2.98
134. Swelling	3	2.56	3.3	2.34	6.2	2.99
135. Dry eyes	2.91	2.58	3.8	2.4	5.6	2.75
136. Menstrual changes	2.9	2.72	3.7	2.48	6.8	3.22
137. Nausea	2.5	2.41	2.5	1.8	5.7	2.91
138. Poor immune system	2.5	2.59	4	3.12	7	2.58
139. Heat flashes	2.4	2.24	3.3	2.42	5.7	3.27
140. Skin allergy	2	2.05	3.4	2.63	5.4	3.54

aPL, antiphospholipid antibodies; APS, antiphospholipid syndrome; HELLP, hemolysis, elevated liver enzyme levels, and low platelet levels; HPRs, nonphysician health professional in rheumatology; MRI, magnetic resonance imaging; sFlt-1/PlGF, soluble fms-like tyrosine kinase-1/placental growth factor.

During the Task Force meeting, votes from the Task Force were counted by the 2 EMEUNET members with the supervision of methodologists. Votes were added to the summary table shared by the convenor with all Task Force members. Voting in favor, voting against, and those abstaining were counted separately in order to determine the denominator. As time was not sufficient to score all items in the first meeting, 2 days later, a second meeting was conducted. After the Task Force meeting, the following decisions were made after discussions with the methodologists and the Task Force members: (1) abstentions in item voting will be reported but deducted from the denominator; (2) the general threshold for including an item will be endorsement by 50% of members; and (3) if there are >25% abstentions in the voting of an item, this item will be excluded. Following a Delphi method, the panel concluded to use a stricter threshold of endorsement by >75% to further reduce the number of items. Using this threshold, 24 items were included.

The steering committee subsequently divided thrombocytopenia into 2 items (severe [platelet count: ≤50,000/µL] and moderate [platelet count: 51,000-100,000/µL]) and split APS nephropathy into 3 items (APS nephropathy-related acute kidney injury, APS nephropathy-related nephrotic-range proteinuria, and APS nephropathy-related nonnephrotic proteinuria). APS nephropathy-related acute kidney injury was defined as a new-onset increase in serum creatinine by at least 1.5-fold the baseline level (based on the definition of acute kidney injury by Kidney Disease: Improving Global Outcomes guidelines). APS nephropathy-related proteinuria can occur independently of serum creatinine levels. The steering committee also divided VTE into provoked and unprovoked and created separate items for venous thromboembolism in 1 territory and VTE in 2 territories, as well as for arterial events in 1 territory and arterial events in 2 territories. With these alterations, the total number of items increased to 30. Items were organized in 2 scales: the TMN scale and the Obstetric scale (Table 5).

### Phase III. Item scoring

In total, 67 physicians among the Task Force members and collaborators participated in the clinical vignette exercise. Two physicians’ ratings were omitted due to extensive missing responses, leaving 65 physicians who provided ratings. Of these, 60% were men, 68% were aged 55 years or older, 60% were from Europe, 55% were rheumatologists, 74% had been in practice for at least 20 years, and 62% treated 10 or more patients with primary APS monthly (Table 3).

**Table 3:** Characteristics of the physicians (*n* = 65) who completed the ranking exercise of clinical vignettes

Age group	
Age 35-44 y	8 (12)
Age 45-54 y	13 (20)
Age 55-64 y	25 (39)
Age 65 y or older	19 (29)
Gender	
Men	39 (60)
Women	25 (39)
Nonbinary	1 (1)
Geographic area	
Europe	39 (60)
North America	13 (20)
Rest of the world	13 (20)
Medical specialty (rheumatology vs others)	
Rheumatologist	36 (55)
Other specialty	29 (45)
Years in practice	
0-9	2 (3)
10-19	14 (22)
20 or more	48 (74)
Missing	1 (1)
Average number of patients seen monthly	
0-4	8 (12)
5-9	14 (22)
10-19	18 (28)
20 or more	23 (35)
Missing	2 (3)

Three versions of real-case vignettes were abstracted for each of the 30 single items identified (24 TMN and 6 Obstetric). Among TMN manifestations, 185 potential combinations of single items, named double manifestations, were identified. Following consensus, the steering committee agreed that 26 of these double manifestations were the most likely to present concurrently, and clinical cases/vignettes with these double manifestations occurring over a 1-month period were also abstracted.

Pooling the ratings of 24 single-item TMN vignettes (and 1 vignette without new manifestations) across physicians resulted in 1625 ratings of how typical these vignettes were in representing patients seen in their practice. Overall, 31% were rated as very typical, 44% were rated as somewhat typical, 22% were rated as not typical, and ratings were missing for 3%. The vignettes most often rated as not typical were those describing rare manifestations: new seizures (57%), new avascular necrosis (49%), new transverse myelitis (44%), new nephrotic-range proteinuria (38%), new adrenal hemorrhage (34%), and new pulmonary hemorrhage (33%). Among 7 obstetric vignettes (pooled *N* = 455), 60% were rated as very typical, 31% as somewhat typical, 6% as not typical, and ratings were missing for 3%.

Crude and adjusted mean PhysGA ratings of the TMN and obstetric vignettes are shown in Table 4. Mean scores of moderate thrombocytopenia and severe thrombocytopenia were significantly different, as were mean scores of nonnephrotic and nephrotic-range proteinuria, and provoked and unprovoked VTE, so these were retained as separate items.

**Table 4:** Physician global assessment ratings of clinical vignettes

Manifestation	Crude mean (0-10)	Least-squares adjusted mean (0-10)	Item score (0-100)
**TMN manifestations**			
No new or worsening manifestations	0.3	0	0
Venous thromboembolism in one territory, unprovoked	7.98	8.52	85
Venous thromboembolism, provoked	6.06	6.60	66
Venous thromboembolism in 2 territories	8.29	8.83	88
Arterial thrombotic event in 1 territory	8.23	8.77	87
Arterial events in 2 territories	8.64	9.18	91
Thrombosis (arterial or venous) in 3 territories	9.04	9.58	95
Livedoid vasculopathy	6.60	7.13	71
Skin ulcer	5.87	6.41	64
Digital ischemia/gangrene	7.72	8.26	82
Livedo racemosa/livedo reticularis	3.43	3.97	39
APS nephropathy-related nonnephrotic range proteinuria	5.43	5.97	59
APS nephropathy-related nephrotic-range proteinuria	6.56	7.10	71
APS nephropathy-related acute kidney injury	6.16	6.70	67
Moderate thrombocytopenia	4.21	4.75	47
Severe thrombocytopenia	6.07	6.61	66
Hemolytic anemia	7.01	7.55	75
Pulmonary hemorrhage	8.77	9.31	93
Adrenal hemorrhage	7.92	8.46	84
Cardiac valve vegetation	6.01	6.55	65
Cardiac valve thickening	4.50	5.04	50
Pulmonary hypertension	6.72	7.26	72
Transverse myelitis	7.55	8.09	80
Seizures/epilepsy	5.58	6.12	61
Avascular necrosis (osteonecrosis)	5.61	6.60	66
**Obstetric manifestations**			
No pregnancy complications	0.4	0	0
New recurrent first-trimester miscarriage	4.63	4.60	46
Fetal loss	7.27	7.25	72
Eclampsia	8.26	8.23	82
Intrauterine growth restriction	6.78	6.75	67
Premature birth due to placental insufficiency	7.24	7.21	72
HELLP syndrome	8.55	8.52	85

**Table 5:** Thrombotic, microvascular, and nonthrombotic (TMN) manifestations, and obstetric manifestations are included in the EULAR Antiphosholipid Syndrome Disease Activity Score (EAPSDAS)

**A. Thrombotic, microvascular, and non-thrombotic (TMN) manifestations (new or worsening manifestations)**
1. Thrombosis (arterial or venous) in 3 territories
2. Pulmonary hemorrhage
3. Arterial thrombosis^a^ in 2 territories
4. Venous thromboembolism^b^ in 2 territories
5. Arterial thrombosis in 1 territory
6. Venous thromboembolism in 1 territory, unprovoked
7. Adrenal hemorrhage
8. Digital ischemia/gangrene
9. Transverse myelitis
10. Hemolytic anemia
11. Pulmonary hypertension
12. Livedoid vasculopathy
13. APS nephropathy-related nephrotic-range proteinuria
14. APS nephropathy-related acute kidney injury
15. Thrombocytopenia, severe (platelet count: ≤50,000/µL)
16. Venous thromboembolism in 1 territory, provoked
17. Avascular necrosis (osteonecrosis)
18. Cardiac valve vegetation
19. Skin ulcers
20. Seizures/epilepsy
21. APS nephropathy-related nonnephrotic proteinuria (>500 mg/24 h)
22. Cardiac valve thickening
23. Thrombocytopenia, moderate (platelet count: 51-100,000/µL)
24. Livedo reticularis or livedo racemosa

**B. Obstetric manifestations (new manifestations)**
HELLP syndrome
Eclampsia or severe pre-eclampsia
Fetal death
Premature birth due to placental insufficiency (with or without severe features):
Intrauterine growth restriction
New recurrent first-trimester miscarriage
No pregnancy complications

HELLP, hemolysis, elevated liver enzyme levels, and low platelet levels.

Provoked venous thromboembolism is a venous thromboembolism that occurs in the setting of a transient risk factor (major or minor) or a persistent risk factor.

a Arterial thrombosis (confirmed by imaging) includes thromboses in coronary arteries, cerebral arteries, or peripheral, splanchnic, retinal arteries, and organ infarcts (eg, kidney, liver, or spleen).

b Venous thromboembolism (confirmed by imaging) includes pulmonary embolism, deep vein thrombosis (legs or arms), thrombosis/occlusion in splanchnic, renal, cerebral, or retinal veins.

We initially rounded the adjusted mean ratings to integers (0-10) to use as item scores. When these scores were assessed in the paired comparison exercise, 259 of 276 pairwise comparisons were consistent with the ranking of means, whereas 17 (6.1%) were inconsistent (eg, mean vignette rating was higher in clinical vignette version A vs B, whereas a higher proportion of physicians rated B higher than A) (Supplementary Table S2). In 14 cases, the inconsistency was the result of the rounding, which made some manifestations ‘equal’ when the mean rating and the pairwise ranking were consistent. When we omitted rounding and increased the sensitivity of scores by multiplying the adjusted means by 10 (resulting in a 0-100 scale), inconsistency remained for only 3 pairwise comparisons. In these cases (digital ischemia/transverse myelitis; avascular necrosis/VTE provoked; avascular necrosis/ severe thrombocytopenia), there were slight true discrepancies between the mean ratings and the pairwise comparison. Of 15 comparisons of Obstetric scale items, only 1 pair was inconsistent due to rounding, which was resolved by changing to a 0-100 scale (Supplementary Table S3).

We compared mean PhyGA ratings between vignettes that described patients with 2 new manifestations (double manifestations) with vignettes that described each new manifestation separately. In this analysis, it was most informative to test whether the score of the double-manifestation vignette was higher than the highest rated of the 2 single-component manifestations (ie, was the PhysGA boosted by the presence of the lower-rated single manifestation). The analysis of the ratings of double manifestations showed no significant enhancement in scores except for 6 of 26 combinations of manifestations, supporting a ‘bump-up’ in these scores (Table 6).

**Table 6:** Associations of double manifestations with the physician global assessment (PhysGA) ratings. Mean change in PhysGA in vignettes with new double manifestations compared with vignettes with the higher of the 2 component manifestations. Manifestation 1 represents the component item with the higher score, whereas manifestation 2 is the component with the lower score. The difference indicates the increase in PhysGA provided by the combination of manifestation 2 with manifestation 1.

Manifestation 1	Manifestation 2	Adjusted mean difference in PhysGA of manifestation 1 with the addition of manifestation 2 (95% CI)	Bump-up value in the final score
Venous thromboembolism unprovoked	Livedo racemosa/livedo reticularis	−0.32 (−0.91 to 0.27)	
Venous thromboembolism unprovoked	Nephrotic-range proteinuria	0.17 (−0.43 to 0.78)	
Venous thromboembolism unprovoked	Renal insufficiency	−0.24 (−0.82 to 0.33)	
Venous thromboembolism unprovoked	Moderate thrombocytopenia	−0.33 (−0.95 to 0.27)	
Venous thromboembolism unprovoked	Cardiac vegetation	0 (−0.68 to 0.68)	
Arterial thrombosis	Skin ulcers	0.12 (−0.39 to 0.64)	
Arterial thrombosis	Livedo racemosa/reticularis	−0.38 (−0.94 to 0.17)	
Arterial thrombosis	Non-nephrotic proteinuria	0.10 (−0.40 to 0.61)	
Arterial thrombosis	Renal insufficiency	−0.04 (−0.632 to 0.54)	
Arterial thrombosis	Moderate thrombocytopenia	−0.15 (−0.69 to 0.38)	
Arterial thrombosis	Cardiac valve vegetation	0.34 (−0.18 to 0.86)	
Arterial thrombosis	Cardiac valve thickening	−0.75 (−1.39 to −0.11)	
Arterial thrombosis	Seizures	−0.01 (−0.52 to 0.49)	
Digital ischemia/gangrene	Skin ulcers	0.06 (−0.49 to 0.61)	
Digital ischemia/gangrene	Livedoid vasculopathy	−0.06 (−0.61 to 0.50)	
Digital ischemia/gangrene	Livedo racemosa/livedo reticularis	−0.21 (−0.79 to 0.37)	
Hemolytic anemia	Moderate thrombocytopenia	0.70 (−0.01 to 1.42)	
Livedoid vasculopathy	Skin ulcers	−0.11 (−0.58 to 0.37)	
Livedoid vasculopathy	Livedo racemosa/livedo reticularis	0 (−0.54 to 0.54)	
Skin ulcers	Livedo racemosa/livedo reticularis	0.60 (0.01-1.19)	6
Skin ulcers	Nonnephrotic proteinuria	1.20 (0.61-1.79)	12
Severe thrombocytopenia	Skin ulcers	1.18 (0.46-1.90)	11
Renal insufficiency	Severe thrombocytopenia	1.54 (0.81-2.27)	15
Renal insufficiency	Nonnephrotic proteinuria	0.57 (−0.20 to 1.34)	
Nephrotic-range proteinuria	Livedo racemosa/livedo reticularis	1.01 (0.30-1.73)	10
Nonnephrotic proteinuria	Moderate thrombocytopenia	1.72 (0.99-2.45)	17

There was discussion about using mean ratings rather than adding regression coefficients to achieve a summary index (as in the Systemic Lupus Erythematosus Disease Activity Index [SLEDAI]). Based on methodologists’ view, this approach would not be supported by the data since we did not have vignettes with more than 2 manifestations, and mostly have vignettes with 1 manifestation, due to the nature of APS. Summing regression-based weights would extend beyond the data in this case.

During the second Task Force meeting, the panel agreed on a 1-month time frame for TMN scale manifestations and the entire pregnancy for the Obstetric scale. The results of the survey that followed the meeting were not conclusive on the arterial and venous thrombosis time interval: 50% of responses were in favor of a 1-month and 50% of >1-month interval. Thus, a vote among the Task Force members was held. All Task Force members, except for the 3 methodologists, participated in the voting, resulting in a total of 21 members. Among them, 78% endorsed a 1-month interval for arterial thrombotic events, and 72% endorsed a 1-month interval for venous thrombotic events.

## DISCUSSION

APS is an autoimmune disorder with diverse TMN and obstetric manifestations, often challenging to manage [[20], [21], [22], [23]]. Augmenting evidence revealed inflammatory, in addition to classic thrombotic, pathways in APS [[24], [25], [26], [27], [28], [29], [30]], pointing to potential new treatment targets [[31], [32], [33], [34], [35], [36], [37]]. Clinical trials are currently examining the safety and efficacy of immunoregulatory treatments in APS patients with TMN and obstetric manifestations [16] and older agents, eg, reassessing the role of hydroxychloroquine [[37], [38], [39]], supporting the need for validated outcome measures.

EAPSDAS was developed as a numerical tool for quantifying and assessing disease activity in APS. One of the project’s strengths is that expert and evidence-based methodology was used, following the main phases of previously published disease activity scores. Another strength is the participation of a large group of APS experts, HPRs, and patients with primary APS across 5 continents. Multiple surveys were launched to generate all possible items related to disease activity in APS and item ranking for identifying the most important ones to be included in EAPSDAS, followed by item scoring using clinical vignettes. Another strength of the score is that it includes diverse clinical manifestations from multiple organ systems on 2 main scales.

Importantly, clinical vignettes were developed based on real-world cases, and 3 different vignette versions for each of the included items were created to avoid bias. In addition, the development of vignettes was based on cases from 3 different units in Europe and 1 from South America, increasing their generalizability. Two real-world clinical vignettes were included in each vignette exercise to familiarise participants with the content of the exercise. Construct validity was supported using both internal and external standards, while interrater reliability of the scales was tested using clinical vignettes. A training document with detailed instructions for the implementation of the score was also developed and provided to construct validity assessors. This, along with a glossary including definitions for each item, is provided as supplemental material in the main manuscript [18] to facilitate the use of EAPSDAS. Those planning to use the score should carefully review both documents before applying the score in a real-life setting.

Several APS-related manifestations were omitted from the final EAPSDAS because they were not ranked highly by the expert panels considering their importance to be included in a disease activity score (eg, migraine, chorea, longitudinal myelitis, and cognitive dysfunction) or their feasibility for use in daily practice (eg, manifestations whose diagnosis and follow-up is based on magnetic resonance imaging or pathology such as myocardial disease) [[40], [41], [42]]. This included some laboratory tests, such as aPL titers/types, and inflammatory markers. Future studies might examine these or other new laboratory markers as measures of APS activity using the EAPSDAS as the standard.

We did not include the presence, type, or intensity of treatment in the measure of disease activity, as doing so could create circularities of reasoning. Thus, the score is not influenced by various types of treatments used for TMN and obstetric APS manifestations (eg, antiplatelets only, anticoagulants with or without antiplatelets, immunosuppressives or targeted treatments), or the intensity of treatment (eg, international normalized ratio [INR] levels). Notably, although ‘low INR’ was nominated for consideration by physicians, health professionals, and patients in the second open-ended survey, it was rated very low priority as indicated by the physicians’, health professionals’, and patients’ mean rankings in the third survey (85th item in the list of 140 items) (Table 2). The EAPSDAS differs from several other familiar disease activity scores (eg, SLEDAI) in that it is a scale rather than an index [43,44]. Indexes are composite scores that sum together (or otherwise integrate) ratings of diverse manifestations. This approach makes sense for a disease such as SLE, in which activity in the skin, joints, vessels, kidneys, serosa, brain, and bone marrow may all occur simultaneously. This approach is less well suited to APS, where activity is manifested most often by single events (eg, a VTE or a stroke). Therefore, we used the scale format for the EAPSDAS.

The development of EAPSDAS will help enable well-designed clinical trials for new treatments in primary APS.

## Editor disclosure

The peer review process did not involve Editorial Board Member Robert Landewé, and the editorial decision-making was led by editors who were not involved in the creation of this manuscript.

## Competing interests

MGT has received grants to the institution from AbbVie, Boehringer Ingelheim, DEMO, Faran, GSK, and UCB and participation in advisory boards from GSK, Lilly, and UCB. AT has participated in advisory boards from UCB and Galapagos. TS received grant support from Roche and personal fees from AbbVie, Roche, Sanofi, Takeda, and Novartis, outside the submitted work. RBML received consulting fees from AbbVie, BMS, Celgene, Eli-Lilly, Galapagos, Gilead, Glaxo-Smith-Kline, Janssen, Merck, Novartis, Pfizer, Roche, and UCB and is the Director of Rheumatology Consultancy BV. MMW is consultant to Advanced Clinical LLC. RC, GJP-E, and SS have nothing to declare.

## Appendix Supplementary materials

Image, application 1

## Footnotes

Supplementary material associated with this article can be found in the online version at doi:10.1016/j.ero.2025.07.009.
