# Supplementary material for: Methodology of the development of the EULAR Antiphospholipid Syndrome Disease Activity Score (EAPSDAS)
Source: EULAR Rheumatol Open. 2025 Nov 19;2(3):100046. doi: 10.1016/j.ero.2025.07.009 (PMC13650349; doi:10.1016/j.ero.2025.07.009)
Supplement: Supplementary file 1 [file mmc1.docx]

**Supplemental material**

**Methods**

1. Search terms for the systematic literature review

2. Clinical vignette examples. Versions A, B, and C of unprovoked venous thromboembolism.

**Results**

1. Supplemental Table 1

2. Supplemental Table 2

3. Supplemental Table 3

**Methods:**

**1. Search terms for the systematic literature review**

Search strategy used for PubMed. This was adapted for Embase.

("Antiphospholipid Syndrome"[Mesh] OR "Antiphospholipid Syndrome*"[tiab] OR "Anti-Phospholipid Syndrome*"[tiab] OR "Anti Phospholipid Syndrome*"[tiab] OR "Hughes Syndrome"[tiab] OR “anti-phospholipid antibody syndrome*”[tiab] OR “antiphospholipid antibody syndrome*”[tiab])

AND (manifest*[tiab] OR criteria[tiab] OR criterion[tiab] OR “extra-criteria”[tiab] OR “non-criteria”[tiab] OR risk*[tiab] OR "associated with"[tiab] OR characterized[tiab] OR characteristic*[tiab] OR diagnosis[tiab] OR diagnostic*[tiab] OR complication*[tiab])

AND ("Case-Control Studies"[Mesh] OR "case-control"[tiab] OR "Cohort Studies"[Mesh] OR "cohort stud*"[tiab] OR "cohort analys*"[tiab] OR "Cross-Sectional Studies"[Mesh] OR “cross-sectional”[tiab] OR "Follow-Up Studies"[Mesh] OR "Longitudinal Studies"[Mesh:NoExp] OR “systematic review*”[ti] OR “meta-analysis”[ptyp] OR “meta-analysis”[ti] OR “meta-analyses”[ti] OR metanalyses[ti] OR metanalysis[ti] OR “meta analyses”[ti] OR "Review"[Publication Type] OR "Systematic Review"[Publication Type] OR ((longitudinal[tiab] OR prospective[tiab] OR “follow up”[tiab] OR followup[tiab] OR followed[tiab] OR observation*[tiab]) AND (study[tiab] OR studies[tiab] OR analys*[tiab] OR cohort*[tiab] OR measured[tiab] OR measurement*[tiab])))

AND english[lang]

NOT ("Animals"[Mesh] NOT ("Animals"[Mesh] AND "Humans"[Mesh]))

NOT (pediatric*[tw] OR children[tiab] OR child[tiab] OR adolescent*[tw] OR newborn*[tw] OR infant*[tiab] OR youth[tiab] OR teen*[tiab] OR "Infant"[Mesh] OR "Child"[Mesh] OR "Adolescent"[Mesh])

NOT ("Case Reports" [Publication Type] OR “case report*”[tiab] OR “case series”[tiab] OR “case study”[tiab] OR “case studies”[tiab])

**2. Clinical vignette examples. Versions A, B, C of Unprovoked Venous Thromboembolism.**

**Version A**

Identification	38-year-old man

APS history	Diagnosed 1 year ago after presenting with deep vein thrombosis and with confirmatory antibodies.

Other medical history	Diabetes treated with insulin.

Current Presentation	1 week of right upper quadrant abdominal pain. Physical examination showed ascites, hepatomegaly and jaundice. Doppler ultrasonography showed hepatic vein thrombus. Diagnosed with Budd-Chiari syndrome.

No provoking risk factors are present.

Labs	CBC: haemoglobin 7.7 g/dL
Urinalysis normal

Serum Creatinine 1.1 mg/dl, aspartate transaminase (AST): 137 IU/L, alanine transaminase (ALT): 82 IU/L, albumin: 2.2g/dl. direct bilirubin: 2.8 mg/dL

**Version B**

Identification	31-year-old woman

APS history	Diagnosed 3 years ago after presenting with early pre-eclampsia (first pregnancy, previous medical history unremarkable) and with confirmatory antibodies.

Other medical history	None.

Current Presentation	3 days of cramping and soreness on her left calf diagnosed with left popliteal deep venous thrombosis. No provoking risk factors are present. Exam is otherwise unremarkable.

Labs	CBC: normal. D-Dimers >X10 normal values

Urinalysis normal

Serum Creatinine 0.71 mg/dl

**Version C**

Identification	43-year-old woman

APS history	Diagnosed 4 years ago after presenting with deep vein thrombosis and with confirmatory antibodies. She also reported pre-eclampsia the past year.

Other medical history	None

Current Presentation	2 days of headache and seizure. Diagnosed with cerebral venous sinus thrombosis. No provoking risk factors are present.

Exam is otherwise unremarkable.

Labs	CBC normal

Urinalysis normal

Serum Creatinine 0.9 mg/dl

**Supplemental Table 1.** Items extracted by the Systematic Literature Review

**Features/domains**

***Classic thrombotic and obstetric APS manifestations (new)***

1. Catastrophic APS

2. Arterial thrombosis

3. Venous thrombosis

4. Deep vein thrombosis

5. Pulmonary embolism

6. Ischemic stroke

7. Splanchnic venous thrombosis

8. Splanchnic artery thrombosis

9. Cerebral venous sinus thrombosis

10. Myocardial infarction

11. Angina

12. Ophthalmic arterial or venous thrombosis

13. Obstetric complications

14. Fetal death unexplained

15. Recurrent first-trimester miscarriages

16. Severe pre-eclampsia/eclampsia

17. Placental insufficiency

***Laboratory features (new)***

18. Thrombocytopenia

19. Hemolytic anemia (autoimmune or microangiopathic)

20. Persistently high/ increase in anti-β2GPI antibody titers

21. Persistently high/ increase in anticardiolipin antibody titers

22. Low complement levels

23. Proteinuria

24. Elevated D-dimers

25. Lupus anticoagulant

26. Hematuria

27. Newly positive aPL

28. Triple aPL positivity

29. Elevation in liver enzymes due to hepatic thrombosis

***Non-criteria manifestations (new onset)***

30. Microangiopathy, microvascular thrombosis, microthrombosis

31. Livedo reticularis/livedo racemosa

32. Skin ulcers

33. Cardiac vegetations

34. Non-infectious endocarditis (Libman-Sacks)

35. APS nephropathy

36. Epilepsy, new seizures

37. Diffuse alveolar hemorrhage

38. Transverse myelitis

39. Chorea

40. Cognitive impairment

41. Livedoid vasculopathy

42. Amaurosis fugax

43. Pulmonary hypertension

44. Transient ischemic attack

45. Migraine

46. Cardiac valve thickening

47. Heart failure due to cardiomyopathy

48. HELLP syndrome

49. Avascular necrosis

50. ItraUterine Growth Restriction (IUGRI)

51. Digital ischemia/gangrene

52. Adrenal hemorrhage

53. Renal insufficiency

54. Sensorineural hearing loss

55. Acute non-infectious endocarditits

56. Guillain-Barre syndrome

57. Memory issues

58. Premature birth due to placental insufficiency

59. Acute organ dysfunction (kidney, heart, lung, CNS)

60. Splinter hemorrhages

61. Budd Chiari syndrome

62. Oligohydramnios

63. Dementia

64. Multiple sclerosis-like symptoms

65. Portal hypertension

66. Longitudinal myelitis

67. Transient global amnesia

**Imaging features**

68. Radiologic signs of thrombosis (cardiac, brain)

69. Multiple sclerosis-like MRI lesions

70. Brain ischemic lesions on MRI

71. Intracardiac thrombi

72. Limb ischemia-peripheral artery

73. Mesenteric ischemia or infarction

74. Cardiac microvascular disease

75. Splenic infarct

**Histologic features**

76. Histologic signs of microthrombi or thrombotic microangiopathy (skin, cardiac, renal)

77. extensive villous ischemia, villitis, and utero-placental vasculitis, multi-focal utero-placental thromboses

APS: antiphospholipid syndrome, aPL: antiphospholipid antibodies, HELLP: Hemolysis, Elevated Liver enzyme levels, and Low Platelet levels, MRI: magnetic resonance imaging

**Supplemental Table 2**. Pairwise comparisons between TMN manifestation item scores and physician rank ordering of items. Results are based on integer item scores (0 – 10). Each cell presents three percentages, in order: percent of physicians ranking the column item higher than the row item; percent of physicians ranking the column item and row item the same; percent of physicians ranking the column item lower than the row item. Green indicates the majority of physicians expressed a rank order that was consistent with the adjusted mean score; yellow indicates the majority of physicians expressed a rank order that was not consistent with the adjusted mean score, before rounding was omitted.

|  | VTE unprovoked | VTE provoked | VTE in two territories | One Arterial event | Arterial events in two territories | Thrombosis in 3 territories | Livedoid vasculopathy | Skin ulcers | Digital ischemia/gangrene | Livedo reticularis | Non-nephrotic proteinuria | Nephrotic range proteinuria |
| --- | --- | --- | --- | --- | --- | --- | --- | --- | --- | --- | --- | --- |
| VTE unprovoked |  | 15/19/66 | 35/45/20 | 35/45/21 | 35/45/22 | 55/39/6 | 14/11/75 | 9/14/77 | 22/43/ 35 | 3/3/94 | 8/18/74 | 23/17/60 |
| VTE provoked |  |  | 69/20/11 | 68/23/9 | 72/22/6 | 85/12/3 | 46/11/43 | 34/24/52 | 62/20/18 | 14/6/80 | 34/17/49 | 46/15/39 |
| VTE in two territories |  |  |  | 37/28/35 | 32/51/17 | 45/45/10 | 5/21/74 | 8/9/83 | 18/34/48 | 2/0/98 | 3/12/85 | 14/25/61 |
| One Arterial event |  |  |  |  | 20/36/43 | 53/35/7 | 8/14/78 | 8/9/83 | 40/45/15 | 0/5/95 | 5/12/65 | 13/20/66 |
| Arterial events in two territories |  |  |  |  |  | 33/57/11 | 2/12/86 | 5/12/83 | 12/33/53 | 0/0/100 | 2/9/89 | 14/17/69 |
| Thrombosis in 3 territories |  |  |  |  |  |  | 0/6/94 | 2/8/91 | 3/34/63 | 0/0/100 | 0/6/94 | 6/15/78 |
| Livedoid vasculopathy |  |  |  |  |  |  |  | 23/29/48 | 70/20/11 | 0/9/91 | 31/14/55 | 48/18/34 |
| Skin ulcers |  |  |  |  |  |  |  |  | 77/18/5 | 12/9/78 | 34/32/34 | 60/18/22 |
| Digital ischemia/gangrene |  |  |  |  |  |  |  |  |  | 0/6/94 | 9/22/70 | 25/22/54 |
| Livedo reticularis |  |  |  |  |  |  |  |  |  |  | 69/11/20 | 83/3/14 |
| Non-nephrotic proteinuria |  |  |  |  |  |  |  |  |  |  |  | 57/26/17 |

|  | Renal insufficiency | Thrombocytopenia, moderate | Thrombocytopenia, severe | Hemolytic anemia | Pulmonary hemorrhage | Adrenal hemorrhage | Cardiac valve vegetation | Cardiac valve thickening | Pulmonary hypertension | Transverse myelitis | Seizures | Avascular necrosis |
| --- | --- | --- | --- | --- | --- | --- | --- | --- | --- | --- | --- | --- |
| VTE unprovoked | 17/17/66 | 6/5/89 | 14/15/71 | 26/23/51 | 60/29/11 | 37/31/32 | 12/20/68 | 6/9/ 85 | 15/23/62 | 38/22/40 | 9/19/72 | 8/15/77 |
| VTE provoked | 42/20/38 | 17/12/71 | 42/23/35 | 60/14/26 | 82/15/3 | 71/12/17 | 42/15/43 | 19/12/69 | 48/14/38 | 63/18/18 | 34/12/54 | 35/14/51 |
| VTE in two territories | 14/20/66 | 5/3/92 | 5/17/78 | 18/28/54 | 43/43/14 | 40/26/34 | 9/12/79 | 5/95/0 | 14/25/61 | 31/28/41 | 6/14/80 | 3/11/86 |
| One Arterial event | 12/14/74 | 2/3/95 | 10/15/73 | 23/20/56 | 55/26/18 | 35/29/35 | 14/9/77 | 8/3/89 | 22/17/62 | 37/15/48 | 9/6/85 | 9/9/82 |
| Arterial events in two territories | 7/17/75 | 0/5/95 | 3/15/82 | 13/22/65 | 42/40/18 | 29/28/43 | 8/17/75 | 0/8/92 | 6/26/68 | 25/29/46 | 6/12/82 | 5/9/86 |
| Thrombosis in 3 territories | 2/14/84 | 0/2/98 | 2/11/88 | 6/25/69 | 18/62/20 | 9/40/51 | 6/9/86 | 0/3/97 | 5/14/82 | 15/31/54 | 3/6/91 | 0/9/91 |
| Livedoid vasculopathy | 37/22/42 | 8/12/80 | 29/26/45 | 54/23/23 | 94/2/5 | 75/12/12 | 34/23/43 | 6/19/75 | 48/22/31 | 72/6/22 | 32/15/52 | 28/22/51 |
| Skin ulcers | 55/12/32 | 16/12/71 | 42/26/32 | 65/17/19 | 94/3/3 | 82/8/11 | 48/20/32 | 18/17/65 | 62/15/23 | 72/11/17 | 40/15/45 | 40/17/43 |
| Digital ischemia/gangrene | 22/18/60 | 3/6/91 | 12/23/65 | 29/29/42 | 69/20/11 | 55/18/26 | 17/14/69 | 5/11/85 | 29/25/46 | 43/26/31 | 14/12/74 | 14/14/72 |
| Livedo reticularis | 83/4/14 | 51/22/28 | 83/3/14 | 88/3/9 | 97/0/3 | 95/2/3 | 80/8/12 | 60/15/25 | 91/3/6 | 86/5/9 | 71/11/18 | 75/6/18 |
| Non-nephrotic proteinuria | 54/18/28 | 20/14/66 | 43/26/31 | 63/23/14 | 95/2/3 | 83/6/11 | 51/12/37 | 26/20/54 | 63/15/22 | 71/22/8 | 42/23/35 | 35/26/38 |
| Nephrotic range proteinuria | 31/23/46 | 14/8/78 | 29/22/49 | 43/25/32 | 80/17/3 | 65/18/17 | 31/15/54 | 14/12/74 | 48/18/34 | 57/25/18 | 23/23/54 | 25/11/64 |
|  | | | | | | | | | | | | |
|  | Renal insufficiency | Thrombocytopenia, moderate | Thrombocytopenia, severe | Hemolytic anemia | Pulmonary hemorrhage | Adrenal hemorrhage | Cardiac valve vegetation | Cardiac valve thickening | Pulmonary hypertension | Transverse myelitis | Seizures | Avascular necrosis |
| Renal insufficiency |  | 23/5/72 | 38/17/45 | 54/23/23 | 82/12/6 | 65/18/17 | 43/11/46 | 20/18/62 | 46/20/34 | 65/18/17 | 34/18/48 | 31/23/46 |
| Thrombocytopenia, moderate |  |  | 75/9/15 | 85/3/12 | 97/0/3 | 91/3/6 | 72/12/15 | 48/20/32 | 77/11/12 | 86/3/11 | 68/12/20 | 69/11/20 |
| Thrombocytopenia, severe |  |  |  | 57/28/15 | 88/9/3 | 75/9/15 | 40/24/35 | 20/20/60 | 49/26/25 | 68/14/18 | 37/20/43 | 28/28/45 |
| Hemolytic anemia |  |  |  |  | 74/20/6 | 57/20/23 | 22/23/55 | 11/9/80 | 31/25/45 | 43/29/28 | 25/9/66 | 17/20/63 |
| Pulmonary hemorrhage |  |  |  |  |  | 11/35/54 | 2/9/89 | 2/2/97 | 4/17/78 | 9/35/55 | 5/6/89 | 5/5/91 |
| Adrenal hemorrhage |  |  |  |  |  |  | 20/12/68 | 0/9/91 | 23/15/62 | 31/26/43 | 12/12/75 | 8/12/80 |
| Cardiac valve vegetation |  |  |  |  |  |  |  | 9/31/60 | 43/25/32 | 68/9/23 | 35/23/42 | 38/22/40 |
| Cardiac valve thickening |  |  |  |  |  |  |  |  | 82/9/9 | 83/8/9 | 54/25/21 | 57/20/23 |
| Pulmonary hypertension |  |  |  |  |  |  |  |  |  | 52/17/31 | 25/18/57 | 18/22/60 |
| Transverse myelitis |  |  |  |  |  |  |  |  |  |  | 12/17/71 | 16/12/72 |
| Seizures |  |  |  |  |  |  |  |  |  |  |  | 34/26/40 |
| Avascular necrosis |  |  |  |  |  |  |  |  |  |  |  |  |

**Supplemental Table 3.** Pairwise comparisons between Obstetric item scores and physician rank ordering of items. Results are based on integer item scores (0 – 10). Each cell presents three percentages, in order: percent of physicians ranking the column item higher than the row item; percent of physicians ranking the column item and row item the same; percent of physicians ranking the column item lower than the row item. Green indicates the majority of physicians expressed a rank order that was consistent with the adjusted mean score; yellow indicates the majority of physicians expressed a rank order that was not consistent with the adjusted mean score, before rounding was omitted.

|  | Fetal death | Severe pre-eclampsia/  Eclampsia | Intrauterine growth restriction | Premature birth due  to placental insufficiency | HELLP syndrome |
| --- | --- | --- | --- | --- | --- |
| New recurrent first trimester miscarriage | 77/20/3 | 86/8/6 | 68/15/17 | 77/12/11 | 91/6/3 |
| Fetal loss |  | 51/29/20 | 21/23/55 | 23/45/32 | 58/25/17 |
| Eclampsia or severe pre-eclampsia |  |  | 8/17/75 | 14/26/60 | 38/42/20 |
| Intrauterine growth restriction |  |  |  | 48/31/21 | 81/11/8 |
| Premature birth due to placental insufficiency |  |  |  |  | 66/25/9 |
| HELLP syndrome |  |  |  |  |  |

HELLP: Hemolysis, Elevated Liver enzyme levels, and Low Platelet levels.
